# Crystal structure, spectroscopic characterization and Hirshfeld surface analysis of aqua­dichlorido­{*N*-[(pyridin-2-yl)methyl­idene]aniline}copper(II) monohydrate

**DOI:** 10.1107/S2056989019017213

**Published:** 2020-01-07

**Authors:** Miguel F. Molano, Vaneza P. Lorett Velasquez, Mauricio F. Erben, Diana L. Nossa González, Alix E. Loaiza, Gustavo A. Echeverría, Oscar E. Piro, Yeny A. Tobón, Karima Ben Tayeb, Jovanny A. Gómez Castaño

**Affiliations:** a Laboratorio de Química Teórica y Computacional, Grupo Química-Física Molecular y Modelamiento Computacional (QUIMOL), Facultad de Ciencias, Universidad Pedagógica y Tecnológica de Colombia, Tunja, Boyacá, 050030, Colombia; b Universidad Antonio Nariño, Facultad de Ciencias, Bogotá, Colombia; c Centro de Química Inorgánica (CEQUINOR), Facultad de Ciencias Exactas, Universidad Nacional de La Plata, C.C. 962, 1900 La Plata, Argentina; d Departamento de Química, Facultad de Ciencias, Pontificia Universidad Javeriana, 110231561 Bogotá, Colombia; e Departamento de Física, Facultad de Ciencias Exactas, Universidad Nacional de La Plata and IFLP(CONICET), C.C. 67, 1900 La Plata, Argentina; f University of Lille, CNRS, UMR 8516, LASIR - Laboratoire de Spectrochimie Infrarouge et Raman, F-59000 Lille, France

**Keywords:** crystal structure, copper(II), five-coordinate complexes, distorted square pyramid

## Abstract

The reaction of *N*-phenyl-1-(pyridin-2-yl)methanimine with copper chloride dihydrate produced the title neutral complex, [CuCl_2_(C_12_H_10_N_2_)(H_2_O)]·H_2_O. The Cu^II^ ion is five-coordinated in a distorted square-pyramidal geometry, in which the two N atoms of the bidentate Schiff base, as well as one chloro and a water mol­ecule, form the irregular base of the pyramidal structure. Meanwhile, the apical chloride ligand inter­acts through a strong hydrogen bond with a water mol­ecule of crystallization. In the crystal, mol­ecules are arranged in pairs, forming a stacking of symmetrical cyclic dimers that inter­act in turn through strong hydrogen bonds between the chloride ligands and both the coordinated and the crystallization water mol­ecules. The mol­ecular and electronic structures of the complex were also studied in detail using EPR (continuous and pulsed), FT–IR and Raman spectroscopy, as well as magnetization measurements. Likewise, Hirshfeld surface analysis was used to investigate the inter­molecular inter­actions in the crystal packing.

## Abstract 2

The title complex contains four distorted square-pyramidal mol­ecules in the asymmetric unit, each of which inter­acts with another mol­ecule located in an adjacent unit cell by means of two hydrogen-bonded water mol­ecules of crystallization, thus forming symmetric dimers that govern the supra­molecular features of the infinite lattice.

## Chemical context   

Cu^II^ ions coordinated by di­imine N-donor ligands (–N=C—C=N–) are of great inter­est since they combine structural flexibility with other desired characteristics, such as ease of preparation, photophysical (Barwiolek *et al.*, 2016 ▸) and photobiological (Banerjee *et al.*, 2016 ▸) properties, and catalytic activity (Dias *et al.*, 2010 ▸), as well as the capability to mimic active protein sites (Gupta & Sutar, 2008 ▸) and stabilize both metal oxidation states common in biological systems. These complexes also exhibit a broad spectrum of pharmacological properties including anti-inflammatory, anti­bacterial, anti­oxidant and anti­metastatic (Chaviara *et al.*, 2005 ▸) activities. In particular, they are promising metallotherapeutic drugs for the treatment of cancer, given their ability to induce apoptosis or generate reactive oxygen species (ROS) in oxidative stress, resulting in DNA damage and strand breaks in cancerous cells (Trudu *et al.*, 2015 ▸).

In particular, bidentate pyridinyl­imine (C_5_H_4_N—CH_2_—NH—C_6_H_5_) and pyridinyl­methyl­amine (C_5_H_4_N–CH=N—C_6_H_5_) Schiff base derivatives have attracted increasing attention because of their close structural relationship with the protein Aβ aggregate *p*-I-stilbene [I-C_6_H_4_—CH=N—C_6_H_4_—*R*, *R* = N(CH_3_)_2_] and thus their potential use for the development of metal chelators for the attenuation of metal-involved neurodegeneration in Alzheimer’s disease (DeToma *et al.*, 2012 ▸). These ligands can therefore act as chemical reagents that can target metal-associated amyloid-β (Aβ) species and modulate metal-induced Aβ aggregation and neurotoxicity *in vitro* and in living cells (Braymer *et al.*, 2012 ▸).

Based on their relevant structural features and promising biological activity, we have begun to explore novel metal complexes coordinated with di­imine ligands (Schiff bases). We report here the synthesis and structural characterization of the complex [Cu(H_2_O)Cl_2_(C_12_H_10_N_2_)]·H_2_O where C_12_H_10_N_2_ = *N*-(pyridin-2-yl­methyl­ene)aniline. This compound is formed by the reaction of copper chloride dihydrate with the C_12_H_10_N_2_ Schiff base to afford bright-green crystals suitable for X-ray diffraction studies.
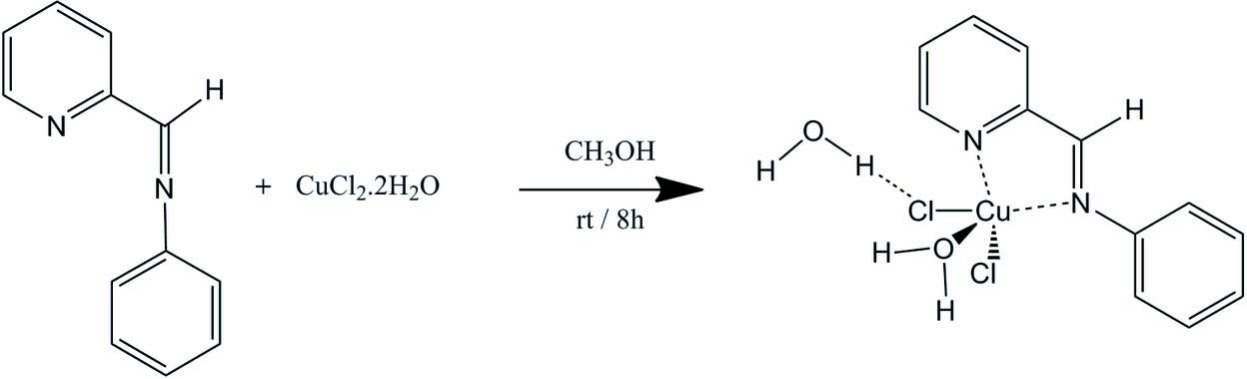


## Structural commentary   

**Figure 1:**
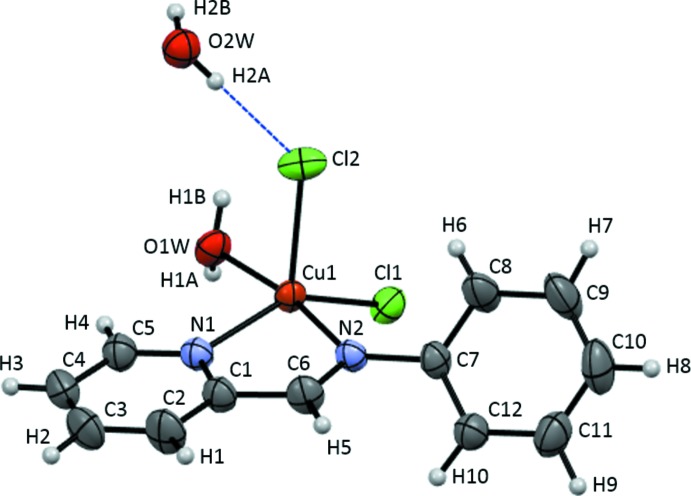
*ORTEP* representation of the title complex with the atom numbering. Displacement ellipsoids are drawn at 50% probability level. The hydrogen bond to the water mol­ecule of crystallization is shown as a dashed blue line.

The title complex crystallizes in the monoclinic space group *P*2_1_/n with *Z* = 4 mol­ecules per unit cell. The Cu^II^ ion is five-coordinated by two nitro­gen atoms from the di­imine ligand and a water mol­ecule in the equatorial position, and two chloro ligands that provide an apical and a pseudo-equatorial coordination, as shown in Fig. 1 ▸. The apical Cu1—Cl2 distance is 0.193 Å longer than that of the non-apical Cu1—Cl1 distance (Table 1 ▸). The apical chloro atom, Cl2, is hydrogen bonded to the water mol­ecule of crystallization, O2*W*—H2*A*⋯Cl2 [2.357 (12) Å; Table 2 ▸]. As a result, the coordination geometry around the Cu^II^ ion is best described as a distorted square-pyramidal structure with a trigonal–bipyramidal component of structural index τ = 0.40 [= (β - α)/60, where β = O1*W*—Cu1—N2 = 169.12 (8)° and α = N1—Cu1—Cl1 = 145.15 (6)°]; for perfect square-pyramidal and trigonal–bipyramidal geom­etries, the values of τ are zero and unity, respectively (Addison *et al.*, 1984 ▸). The two N—Cu distances, however, differ by only 0.019 Å. The trigonal-component axial compression (%TC) is −3.22 [%TC = 100(*B*—*D*)/*B*, where *B* is the Cu1—O1*W* distance and *D* the Cu1—N1 distance; Addison *et al.*, 1984 ▸]. Intra­molecular C8—H6⋯Cl2 and C5—H4⋯O1*W* hydrogen bonds occur (Table 2 ▸).

## Supra­molecular features   

**Figure 2:**
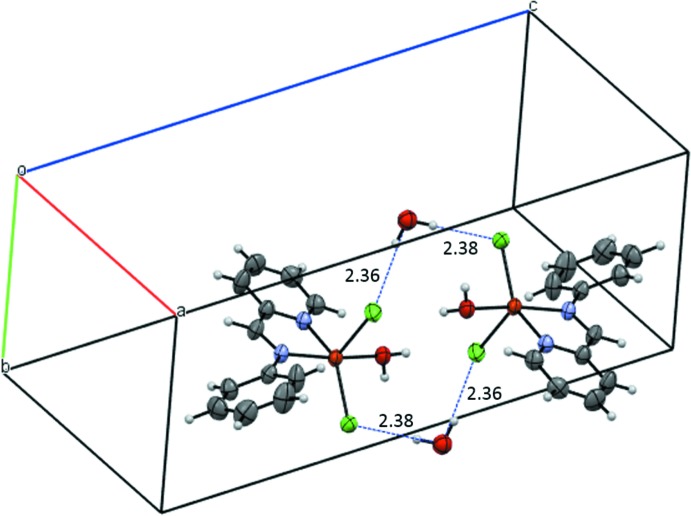
Symmetric cyclic dimers in the crystal structure of the title complex formed by dual Cl⋯H—O—H⋯Cl inter­actions (dotted blue lines) between the chlorine ligands and the water mol­ecules.

**Figure 3:**
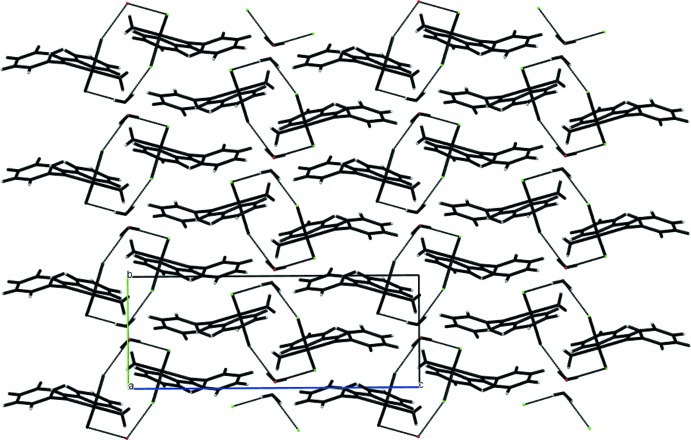
The crystal packing in a view along the ***a*** **+** ***b*** vector showing the stacking of symmetric cyclic dimers.

As expected, both the water mol­ecule of crystallization and the aqua ligand play a significant role in the crystal packing of the complex. This is governed by the presence of symmetric cyclic dimers formed between complex mol­ecules in adjacent unit cells along the *a*-axis direction (see Fig. 2 ▸). Each dimer comprises two water mol­ecules of crystallization, each of which links the two complex monomers by two different hydrogen bonds, one with the apical Cl2 ligand [2.357 (12) Å] and the second with the non-apical Cl1 ligand [2.384 (13) Å]. The dimers are stacked alternately in the *b*-axis direction, forming a wave-like arrangement as shown in Fig. 3 ▸. Each dimer inter­acts with two other dimers through two different hydrogen bonds. One of these [2.85 (3) Å], is formed between the apical chlorine Cl2 and the aromatic hydrogen H10 in the *ortho* position, while the second [2.56 (3) Å], is formed between the non-apical chlorine Cl1 and the aliphatic hydrogen H5 of the CH group. In addition, each apical chloro ligand Cl2 inter­acts with the hydrogen atom H1*B* of the aqua O atom O1*W* of a third dimer by means of a shorter hydrogen bond [2.347 (15) Å], with the other hydrogen atom H2*A* of the aqua ligand, forming a quite short hydrogen bond [1.795 (11) Å] with the oxygen atom O2*W* of the water mol­ecule of crystallization.

## Hirshfeld surface analysis   

**Figure 4:**
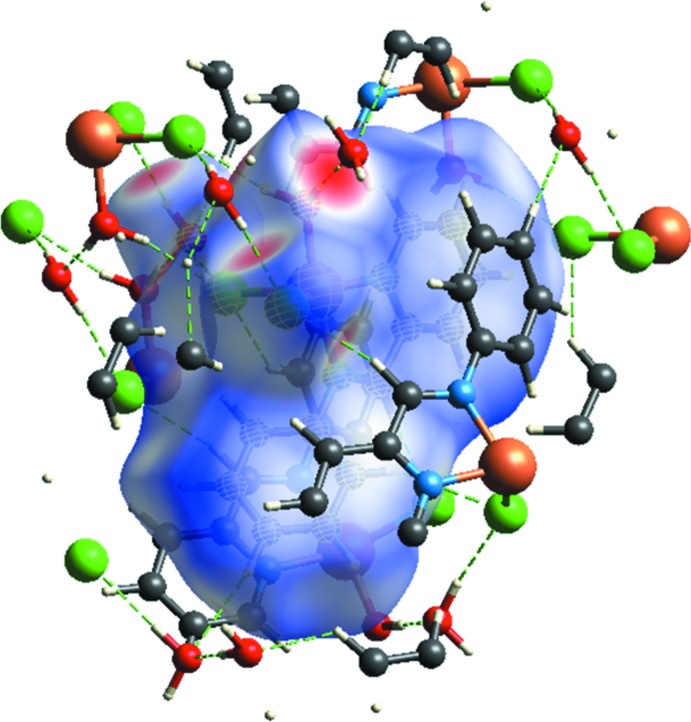
Hirshfeld surface of the title complex mapped over electrostatic potential in the range 0.5 to 1.5 atomic units.

**Figure 5:**
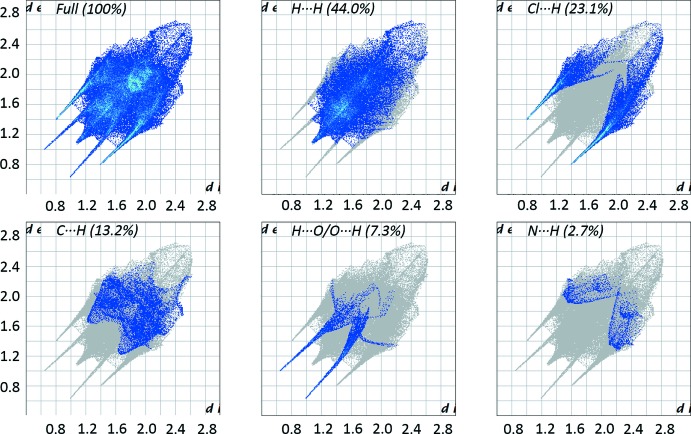
Two-dimensional fingerprint plots for all contacts and delineated into H⋯H, Cl⋯H, C⋯H, H⋯O/O⋯H, and N⋯H contacts in the title complex.

In order to investigate and visualize the role of weak inter­molecular inter­actions, a Hirshfeld surface (HS) analysis (Spackman & Jayatilaka, 2009 ▸) was carried out and the associated two-dimensional fingerprint plots (McKinnon *et al.*, 2007 ▸) generated using *CrystalExplorer17.5* (Turner *et al.*, 2017 ▸). The three dimensional *d*_norm_ surface of the title compound using a standard surface resolution with a fixed colour scale of 0.5 to 1.5 a.u. is shown in Fig. 4 ▸. The darkest red spots on this surface correspond to the H_2_O⋯H—O and H—O—H⋯Cl hydrogen bonds resulting from the inter­actions between the water mol­ecule of crystallization and the coordinated water and chlorine, respectively. The fingerprint plots in Fig. 5 ▸, for all inter­actions in the title compound, and those delineated into H⋯H, Cl⋯H, C⋯H, H⋯O/O⋯H and N⋯H contacts, exhibit four pseudo-symmetric long sharp spikes characteristic of strong hydrogen bonds and one spike in the *d_e_* and *d_i_* diagonal axes associated with H⋯H inter­actions. The greatest contribution to the HS is from H⋯H inter­actions (44.0%), which are represented by a distinctive sharp spike in the region *d_e_* = *d_i_* ≃ 1.5 Å. The Cl⋯H contacts make a 23.1% contribution to the HS and are represented by a pair of sharp spikes in the region *d_e_* + *d_i_* ≃ 2.2 Å. The C⋯H contacts (13.2% contribution) are observed as two wide contour signals in the region *d_e_* + *d_i_* ≃ 3.0 Å. The N⋯H contacts (2.7%) are represented by two signals with thick edges in the region *d_e_* + *d_i_* ≃ 3.3 Å. The O⋯H contacts are represented by two sharp spikes in the region *d_e_* + *d_i_* ≃ 1.6 Å, which indicates a clear formation of hydrogen bonds.

## CW-EPR/Pulsed-EPR and PPMS characterization   

**Figure 6:**
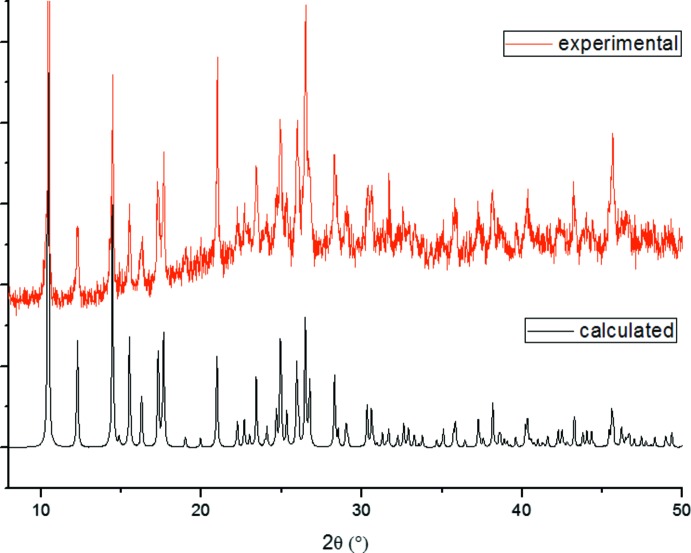
Comparison between the experimental and theoretical powder diffractogram for the title complex. The calculated diffractogram was simulated using *Mercury* software (Macrae *et al.*, 2020 ▸) from the CIF file.

In order to obtain in-depth information on the spin properties of this unpaired spin complex (*d* ^9^, 2*S* + 1 = 2), electron paramagnetic resonance (EPR) continuous-wave (CW) experiments were performed on a X-Band Bruker ELEXSYS E500 spectrometer operating at 9.8 GHz. The powdered sample was inserted in a quartz tube and the spectra were recorded at room temperature and 100 K under non-saturated conditions: microwave power of 0.63 mW and modulation amplitude of 2 G. Pulsed EPR was studied at 5 K with a Bruker ELEXSYS E580 spectrometer equipped with a helium flow cryostat. Two-pulse echo field sweep acquisitions were performed using a standard Hahn echo sequence 90 − τ − 180 with a 90° pulse length of 16 ns and τ value of 172 ns. The HYperfine Sublevel CORrElation spectroscopy (HYSCORE) experiments (Höfer *et al.*, 1986 ▸) were recorded with 256 × 256 data points for both the *t*_1_ and *t*_2_ time domains, 90° pulse length of 16 ns and an echo delay of 172 and 200 ns. The obtained HYSCORE spectra are composed of two quadrants: the first quadrant (+,−) where *A* > 2*n_I_* (*n_I_* being the nuclear frequency) corresponds to the strong hyperfine coupling *A* between the *I* nucleus and the unpaired electron and the second quadrant (+,+) where *A* < 2*n_I_* corresponds to weaker inter­actions. Magnetization measurements were performed with a physical property measurement system (PPMS) Quantum Design Dynacool of 9 T and the vibrating sample magnetometer (VSM) option. To verify that both samples, *i.e*. powder and crystal, correspond to the same compound, a comparison between the X-ray powder diffraction pattern and the simulated single X-ray diffraction pattern is presented in Fig. 6 ▸.

**Figure 7:**
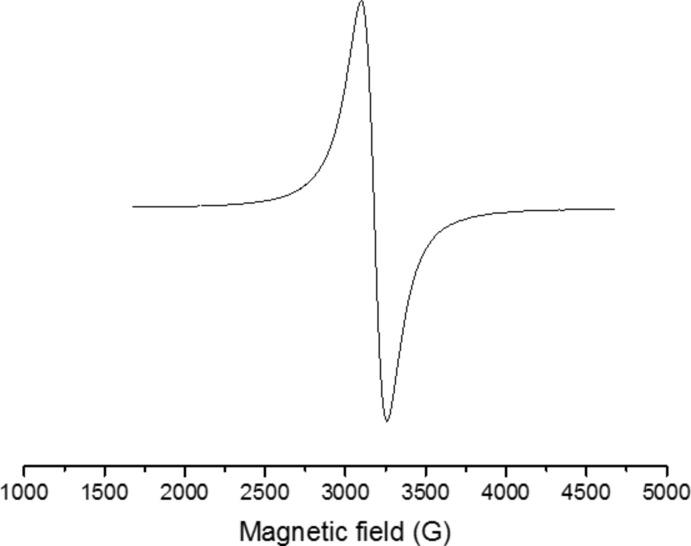
CW–EPR spectrum for the title complex measured at 100 K.

**Figure 8:**
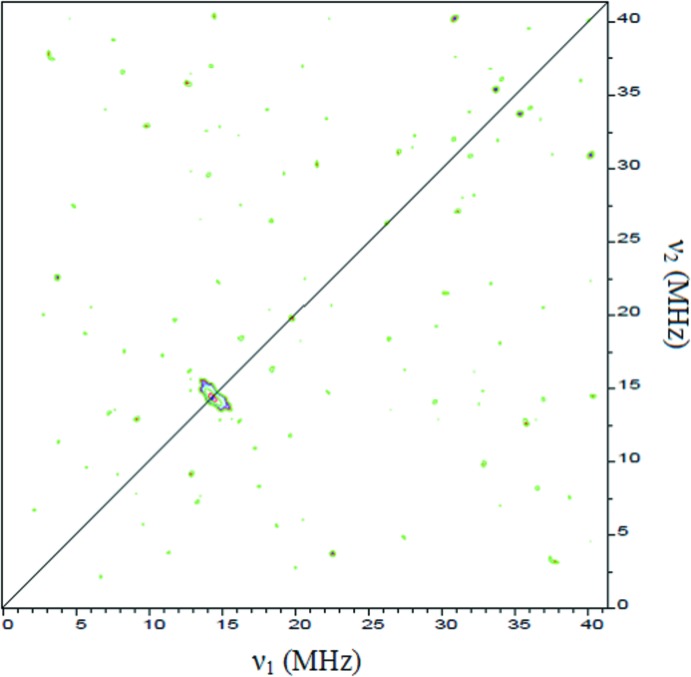
Two-dimensional HYSCORE spectrum of the title complex recorded at 5 K.

A strong isotropic signal characteristic of Cu^II^ was detected by EPR spectroscopy with *g*_iso_ = 2.13 and a line width of 130 G at room temperature. No sign of anisotropic behaviour was detected in the low-temperature continuous wave EPR spectra recorded at 100 K (Fig. 7 ▸) and 8 K. Further efforts to reveal possible minor anisotropic behavior through *Q*-band (34 GHz) low-temperature (8 K) measurements still showed a single strong band characteristic of the Cu^II^ ion in an isotropic environment. The isotropic signal was so dominant in the powder spectrum that the anisotropic features were invisible. A similar observation was made by Xavier & Murugesan (1998 ▸). 98 mg of Cu^II^ were qu­anti­fied in the complex sample by continuous wave EPR using copper sulfate with a known mass as standard. To obtain more information about the surroundings of the copper(II) centre, a pulsed EPR experiment was performed using HYSCORE. In quadrant (+,+), a unique signal characteristic of hydrogen was detected with a Larmor frequency of 14.6 MHz corresponding to a weak inter­action between the copper unpaired electron and the hydrogen nucleus (Fig. 8 ▸). Inter­actions between the copper and the nitro­gen atoms were not observed. It is probable that the inter­action is too strong to be detected by the HYSCORE sequence.

**Figure 9:**
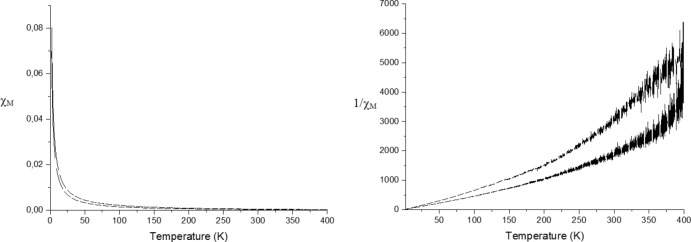
Temperature dependence of molar magnetic susceptibility χ_*M*_ and inverse susceptibility 1/χ_*M*_.

**Figure 10:**
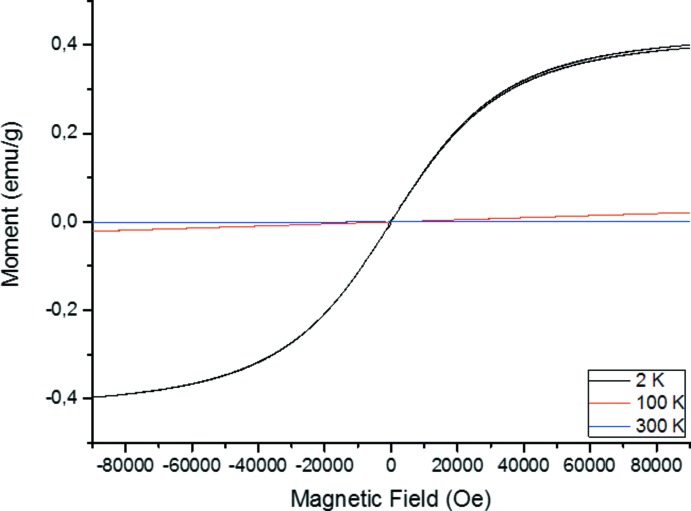
Dependence of magnetization on the magnetic field at 2, 100 and 300 K.

Magnetic susceptibility measurements were performed to verify the nature of coupling between the cupric ions. The temperature dependence of the molar magnetic susceptibility χ_*M*_ and the corresponding inverse susceptibility 1/χ_*M*_ measured at a magnetic field of 0.1 T in the temperature range of 2–400 K is shown in Fig. 9 ▸. Fig. 10 ▸ shows the dependence of magnetization on the magnetic field at 2 K, 100 K and 300 K. At higher temperature, the magnetization manifest Curie–Weiss-like behaviour. The magnetization curves of the sample have features typical of a paramagnetic contribution between magnetic centers

## Database survey   

A survey of the Cambridge Structural Database (CSD, Version 5.40, Oct 2019; Groom *et al.*, 2016 ▸) reveals that crystal structures have been reported for coordinated Cu^II^ and Zn^II^ complexes containing *N*-(pyridin-2-ylmeth­yl)aniline and its deprotonated form *N*-(pyridin-2-yl­methyl­ene)aniline, respectively (Braymer *et al.*, 2012 ▸). For the former complex, [Cu(C_12_H_12_N_2_)Cl_2_], a nearly square-planar geometry between the bidentate Schiff base and two chloro ligands was reported; while for the latter, [Zn(C_12_H_10_N_2_)Cl_2_], a distorted tetra­hedral geometry was observed. A detailed revision of the CIF file reported for the [Cu(C_12_H_12_N_2_)Cl_2_] complex, however, reveals that the crystal packing of this compound can be best described as comprising polymeric chains of complex units consisting of slightly distorted square-pyramidal [(μ)Cu-Cu(C_12_H_12_N_2_)Cl_2_] where the apical position is occupied by a bridged Cu^II^ ion.

## Synthesis and crystallization   

The *N*-(pyridin-2-yl­methyl­ene)aniline ligand, C_12_H_10_N_2_, was prepared by condensation reaction between 2-pyridine­aldehyde (Sigma–Aldrich, 99%) and aniline (Sigma–Aldrich, 99%) in dry methanol (Merck, HPLC grade) at reflux temperature for 4 h under atmospheric pressure and constant stirring. The stoichiometry used in this reaction was 1:1 mmol. The released water vapour was prevented from returning to the reaction vessel by placing a condensation trap containing methanol in the lower base of the reflux column. No byproduct was formed during the reaction. The purity and mol­ecular weight of the ligand was confirmed by GC/MS spectrometry using an Agilent 6850 series II gas chromatograph (CG) coupled to an Agilent 5975B VL MSD mass spectrometer (MS) equipped with a split/splitess injection port (533 K, split ratio 15:1), with an Agilent 6850 series automatic injector and an Agilent 19091S-433E HP-5MS column.

**MS** (*m*/*z* ratio, %): C_12_H_10_N_2_ (182.1, 33.09%); C_12_H_9_N_2_ (181.1, 100%); C_5_H_6_ (77.05, 35.85%); C_5_H_4_N (78.05, 12.98%); C_6_H_5_N_2_ (105.05, 9.11%); C_6_H_5_N (91.05, 4.25%).

The title Cu^II^ complex was prepared by reacting the C_12_H_10_N_2_ ligand with copper chloride dihydrate, CuCl_2_·2H_2_O (Merck, 99.9%), in dry methanol for 8 h at room temperature, under atmospheric pressure and constant stirring. The Cu^II^ complex precipitated in methanol as a green solid, which was then separated from the solvent by rotoevaporation. Crystallization was carried out from a saturated solution of the Cu^II^ complex in methanol at 313 K, which was allowed to cool to room temperature and then hexane was added until reaching a 1:1 methanol/hexane ratio, followed by storage at 277 K. Crystals of the title Cu^II^ complex were separated by subtled deca­ntation and evaporation of the solvents at room temperature.

An infrared (IR) spectrum in attenuated total reflectance (ATR) was acquired from a ground crystal using a Shimadzu Prestigie-21 spectrophotometer with Fourier Transform (FTIR), equipped with a Michelson-type inter­ferometer, a KBr/Ge beam-splitter, a ceramic lamp and DLATGS detector. The FTIR spectrum was measured in the 4000–500 cm^−1^ range with a resolution of 3.0 cm^−1^ and 30 scans. Likewise, Raman spectra of the title complex were obtained using a LabRAM HR confocal Raman microscope (Horiba Scientific) operating in a backlit orientation and equipped with a cryogenic detector and laser lines of 473, 532 and 633 nm of 18, 30 and 17 mW maximum power, respectively. The micro-Raman spectra of the complex were taken through an Olympus 50× long-working-distance microscope objective (NA = 0.5, WD = 10.6 mm), in the range from 3500 to 100 cm^−1^, with a resolution of 4 cm^−1^ and a laser power of around 3.0 mW.

**IR** (ATR, cm^−1^): 3067 C—H Pyr, 3213 C—H Ph, 1601 C=N Schiff B., 1540 C=N Pyr, 1434 C—H, 693 Cu—N. **Raman** (cm^−1^), 1601 C=N Schiff B., 1540 C=N Pyr, 552 Cu—N, 693 Cu—N, 411 Cu—O, 272 Cu—Cl.

## Refinement   

Crystal data, data collection and structure refinement details are summarized in Table 3 ▸. The hydrogen atoms were found in difference-Fourier maps. The O⋯H and H⋯H distances in both water molecules were fixed at 0.86 (1) and 1.36 (2) Å, respectively.

## Supplementary Material

Crystal structure: contains datablock(s) I, global. DOI: 10.1107/S2056989019017213/ex2027sup1.cif

CCDC reference: 1974364

Additional supporting information:  crystallographic information; 3D view; checkCIF report

## Figures and Tables

**Table 1:** Selected geometric parameters (Å, °)

N1—Cu1	2.046 (2)	Cl1—Cu1	2.2744 (7)
N2—Cu1	2.0345 (19)	Cl2—Cu1	2.4673 (7)
Cu1—O1*W*	1.9821 (18)	C7—N2	1.431 (3)
			
O1*W*—Cu1—N2	169.12 (8)	N1—Cu1—Cl1	145.15 (6)
O1*W*—Cu1—N1	88.66 (8)	O1*W*—Cu1—Cl2	88.86 (6)
N2—Cu1—N1	80.47 (8)	N2—Cu1—Cl2	94.66 (6)
O1*W*—Cu1—Cl1	92.92 (6)	N1—Cu1—Cl2	106.80 (6)
N2—Cu1—Cl1	95.76 (6)	Cl1—Cu1—Cl2	108.04 (3)

**Table 2:** Hydrogen-bond geometry (Å, °)

*D*—H⋯*A*	*D*—H	H⋯*A*	*D*⋯*A*	*D*—H⋯*A*
O1*W*—H1*A*⋯O2*W* ^i^	0.85 (1)	1.80 (1)	2.640 (3)	171 (3)
O1*W*—H1*B*⋯Cl2^ii^	0.86 (1)	2.35 (2)	3.1740 (19)	162 (3)
O2*W*—H2*A*⋯Cl2	0.85 (1)	2.36 (1)	3.199 (2)	171 (3)
O2*W*—H2*B*⋯Cl1^ii^	0.85 (1)	2.38 (1)	3.215 (2)	167 (3)
C5—H4⋯O1*W*	0.95 (2)	2.47 (2)	2.999 (3)	115.3 (18)
C6—H5⋯Cl1^iii^	1.00 (3)	2.56 (3)	3.540 (3)	169 (2)
C8—H6⋯Cl2	0.87 (3)	2.95 (3)	3.707 (3)	147 (2)
C12—H10⋯Cl2^iv^	0.95 (3)	2.85 (3)	3.667 (3)	145 (2)

Symmetry codes: (i) 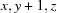; (ii) 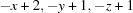; (iii) 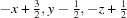; (iv) 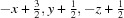.

**Table 3:** Experimental details

Crystal data	
Chemical formula	[CuCl_2_(C_12_H_10_N_2_)(H_2_O)]·H_2_O
*M*_r_	352.69
Crystal system, space group	Monoclinic, *P*2_1_/*n*
Temperature (K)	297
*a*, *b*, *c* (Å)	9.3322 (2), 7.7341 (2), 20.1143 (4)
β (°)	90.002 (2)
*V* (Å^3^)	1451.77 (6)
*Z*	4
Radiation type	Mo *K*α
μ (mm^−1^)	1.87
Crystal size (mm)	0.24 × 0.12 × 0.07

Data collection	
Diffractometer	Rigaku Xcalibur Eos Gemini
Absorption correction	Multi-scan (*CrysAlis PRO*; Rigaku OD, 2015 ▸)
*T*_min_, *T*_max_	0.888, 1.000
No. of measured, independent and observed [*I* > 2σ(*I*)] reflections	6613, 3143, 2380
*R*_int_	0.027
(sin θ/λ)_max_ (Å^−1^)	0.678

Refinement	
*R*[*F* ^2^ > 2σ(*F* ^2^)], *wR*(*F* ^2^), *S*	0.035, 0.080, 1.04
No. of reflections	3143
No. of parameters	228
No. of restraints	6
H-atom treatment	All H-atom parameters refined
Δρ_max_, Δρ_min_ (e Å^−3^)	0.26, −0.39

Computer programs: *CrysAlis PRO* (Rigaku OD, 2015 ▸), *SHELXT* (Sheldrick, 2015*a* ▸), *SHELXL2014* (Sheldrick, 2015*b* ▸), *ORTEP-3 for Windows* (Farrugia, 2012 ▸) and *Mercury* (Macrae *et al.*, 2020 ▸).

## supplementary crystallographic information

### Crystal data

**Table:**

[CuCl_2_(C_12_H_10_N_2_)(H_2_O)]·H_2_O	*F*(000) = 716
*M**_r_* = 352.69	*D*_x_ = 1.614 Mg m^−^^3^
Monoclinic, *P*2_1_/*n*	Mo *K*α radiation, λ = 0.71073 Å
*a* = 9.3322 (2) Å	Cell parameters from 2118 reflections
*b* = 7.7341 (2) Å	θ = 4.0–27.5°
*c* = 20.1143 (4) Å	µ = 1.87 mm^−^^1^
β = 90.002 (2)°	*T* = 297 K
*V* = 1451.77 (6) Å^3^	Fragment, green
*Z* = 4	0.24 × 0.12 × 0.07 mm

### Data collection

**Table:**

Rigaku Xcalibur Eos Gemini diffractometer	3143 independent reflections
Radiation source: fine-focus sealed X-ray tube, Enhance (Mo) X-ray Source	2380 reflections with *I* > 2σ(*I*)
Graphite monochromator	*R*_int_ = 0.027
Detector resolution: 16.0604 pixels mm^-1^	θ_max_ = 28.8°, θ_min_ = 3.3°
ω scans	*h* = −12→10
Absorption correction: multi-scan (CrysAlis PRO; Rigaku OD, 2015)	*k* = −10→9
*T*_min_ = 0.888, *T*_max_ = 1.000	*l* = −26→26
6613 measured reflections	

### Refinement

**Table:**

Refinement on *F*^2^	Primary atom site location: dual
Least-squares matrix: full	Secondary atom site location: difference Fourier map
*R*[*F*^2^ > 2σ(*F*^2^)] = 0.035	Hydrogen site location: difference Fourier map
*wR*(*F*^2^) = 0.080	All H-atom parameters refined
*S* = 1.04	*w* = 1/[σ^2^(*F*_o_^2^) + (0.031*P*)^2^ + 0.1405*P*] where *P* = (*F*_o_^2^ + 2*F*_c_^2^)/3
3143 reflections	(Δ/σ)_max_ = 0.001
228 parameters	Δρ_max_ = 0.26 e Å^−^^3^
6 restraints	Δρ_min_ = −0.39 e Å^−^^3^

### Special details

**Table:**

Geometry. All esds (except the esd in the dihedral angle between two l.s. planes) are estimated using the full covariance matrix. The cell esds are taken into account individually in the estimation of esds in distances, angles and torsion angles; correlations between esds in cell parameters are only used when they are defined by crystal symmetry. An approximate (isotropic) treatment of cell esds is used for estimating esds involving l.s. planes.

### Fractional atomic coordinates and isotropic or equivalent isotropic displacement parameters (Å^2^)

**Table:**

	*x*	*y*	*z*	*U*_iso_*/*U*_eq_	
C1	0.5545 (3)	0.5653 (4)	0.33174 (13)	0.0375 (6)	
C2	0.4122 (3)	0.5200 (5)	0.32604 (16)	0.0548 (9)	
C3	0.3235 (3)	0.5432 (5)	0.37988 (16)	0.0551 (9)	
C4	0.3789 (3)	0.6103 (4)	0.43668 (16)	0.0482 (8)	
C5	0.5233 (3)	0.6512 (4)	0.43961 (14)	0.0409 (7)	
C6	0.6568 (3)	0.5473 (4)	0.27790 (13)	0.0399 (7)	
C7	0.8855 (3)	0.5724 (3)	0.23313 (12)	0.0340 (6)	
C8	1.0201 (3)	0.5067 (5)	0.24304 (15)	0.0538 (9)	
C9	1.1138 (4)	0.4932 (6)	0.18965 (17)	0.0664 (11)	
C10	1.0737 (4)	0.5471 (5)	0.12820 (17)	0.0616 (10)	
C11	0.9387 (4)	0.6116 (5)	0.11776 (15)	0.0569 (9)	
C12	0.8442 (3)	0.6267 (4)	0.17029 (13)	0.0432 (7)	
N1	0.6108 (2)	0.6281 (3)	0.38848 (10)	0.0326 (5)	
N2	0.7884 (2)	0.5841 (3)	0.28799 (10)	0.0305 (5)	
Cl1	1.01089 (7)	0.84014 (9)	0.35841 (3)	0.03986 (18)	
Cl2	0.93385 (8)	0.37596 (9)	0.41578 (4)	0.0452 (2)	
Cu1	0.82853 (3)	0.65623 (4)	0.38345 (2)	0.03006 (11)	
O1W	0.8277 (2)	0.7241 (3)	0.47848 (9)	0.0375 (4)	
H1A	0.833 (3)	0.8331 (13)	0.4836 (13)	0.040 (9)*	
H1B	0.896 (3)	0.679 (3)	0.5008 (16)	0.089 (13)*	
O2W	0.8297 (2)	0.0581 (3)	0.50539 (11)	0.0439 (5)	
H2A	0.854 (3)	0.136 (3)	0.4777 (11)	0.072 (12)*	
H2B	0.883 (3)	0.074 (4)	0.5390 (10)	0.082 (12)*	
H1	0.383 (3)	0.474 (4)	0.2868 (14)	0.047 (8)*	
H2	0.231 (3)	0.523 (4)	0.3756 (15)	0.064 (10)*	
H3	0.326 (3)	0.634 (4)	0.4741 (14)	0.049 (9)*	
H4	0.564 (3)	0.698 (3)	0.4789 (12)	0.033 (7)*	
H5	0.615 (3)	0.502 (4)	0.2358 (14)	0.051 (8)*	
H6	1.041 (3)	0.471 (4)	0.2830 (15)	0.056 (9)*	
H7	1.199 (4)	0.443 (4)	0.1993 (16)	0.074 (11)*	
H8	1.135 (4)	0.538 (5)	0.0938 (18)	0.082 (12)*	
H9	0.908 (4)	0.652 (4)	0.0729 (17)	0.079 (11)*	
H10	0.751 (3)	0.675 (3)	0.1664 (13)	0.042 (8)*	

### Atomic displacement parameters (Å^2^)

**Table:**

	*U*^11^	*U*^22^	*U*^33^	*U*^12^	*U*^13^	*U*^23^
C1	0.0279 (13)	0.0484 (17)	0.0362 (14)	−0.0014 (12)	−0.0025 (11)	−0.0019 (13)
C2	0.0303 (16)	0.084 (3)	0.0495 (18)	−0.0072 (16)	−0.0085 (14)	−0.0086 (19)
C3	0.0248 (15)	0.075 (3)	0.066 (2)	−0.0036 (16)	−0.0003 (15)	0.0034 (18)
C4	0.0336 (16)	0.059 (2)	0.0521 (19)	0.0092 (14)	0.0097 (15)	0.0070 (17)
C5	0.0377 (16)	0.0492 (18)	0.0359 (15)	0.0030 (13)	0.0033 (12)	−0.0021 (14)
C6	0.0345 (15)	0.0519 (18)	0.0334 (14)	−0.0035 (13)	−0.0063 (12)	−0.0098 (14)
C7	0.0317 (14)	0.0413 (15)	0.0290 (13)	0.0008 (12)	0.0013 (11)	−0.0093 (12)
C8	0.0375 (17)	0.089 (3)	0.0347 (16)	0.0156 (17)	−0.0055 (13)	−0.0090 (18)
C9	0.0364 (18)	0.105 (3)	0.057 (2)	0.021 (2)	0.0034 (16)	−0.018 (2)
C10	0.050 (2)	0.088 (3)	0.0474 (19)	−0.0040 (19)	0.0170 (16)	−0.022 (2)
C11	0.063 (2)	0.073 (2)	0.0345 (16)	−0.0011 (18)	0.0084 (15)	0.0008 (17)
C12	0.0407 (17)	0.0539 (19)	0.0350 (15)	0.0063 (15)	0.0014 (13)	−0.0003 (14)
N1	0.0291 (11)	0.0379 (13)	0.0308 (11)	0.0014 (10)	−0.0016 (9)	−0.0009 (10)
N2	0.0255 (11)	0.0380 (12)	0.0280 (11)	0.0019 (9)	−0.0026 (9)	−0.0033 (10)
Cl1	0.0365 (4)	0.0499 (4)	0.0331 (3)	−0.0119 (3)	−0.0039 (3)	0.0058 (3)
Cl2	0.0549 (4)	0.0354 (4)	0.0453 (4)	−0.0004 (3)	−0.0182 (3)	0.0042 (3)
Cu1	0.02761 (18)	0.0388 (2)	0.02381 (17)	−0.00335 (14)	−0.00251 (12)	−0.00170 (13)
O1W	0.0409 (11)	0.0437 (13)	0.0280 (9)	−0.0050 (10)	−0.0040 (8)	−0.0017 (9)
O2W	0.0431 (12)	0.0488 (13)	0.0399 (11)	−0.0023 (10)	−0.0004 (10)	−0.0011 (11)

### Geometric parameters (Å, º)

**Table:**

C1—N1	1.347 (3)	C8—C9	1.389 (4)
C1—C2	1.378 (4)	C9—C10	1.357 (5)
C1—C6	1.450 (4)	C10—C11	1.371 (5)
C2—C3	1.375 (4)	C11—C12	1.381 (4)
C3—C4	1.357 (4)	N1—Cu1	2.046 (2)
C4—C5	1.386 (4)	N2—Cu1	2.0345 (19)
C5—N1	1.325 (3)	Cu1—O1W	1.9821 (18)
C6—N2	1.276 (3)	Cl1—Cu1	2.2744 (7)
C7—C12	1.387 (4)	Cl2—Cu1	2.4673 (7)
C7—C8	1.369 (4)	C7—N2	1.431 (3)
			
N1—C1—C2	122.6 (3)	C1—N1—C5	117.8 (2)
N1—C1—C6	114.2 (2)	C1—N1—Cu1	112.58 (17)
C2—C1—C6	123.2 (2)	C5—N1—Cu1	129.49 (17)
C1—C2—C3	118.8 (3)	C6—N2—C7	118.2 (2)
C4—C3—C2	118.9 (3)	C6—N2—Cu1	112.85 (18)
C3—C4—C5	119.6 (3)	C7—N2—Cu1	128.92 (15)
N1—C5—C4	122.4 (3)	O1W—Cu1—N2	169.12 (8)
N2—C6—C1	119.5 (2)	O1W—Cu1—N1	88.66 (8)
C12—C7—C8	120.0 (3)	N2—Cu1—N1	80.47 (8)
C12—C7—N2	120.5 (2)	O1W—Cu1—Cl1	92.92 (6)
C8—C7—N2	119.5 (2)	N2—Cu1—Cl1	95.76 (6)
C7—C8—C9	119.5 (3)	N1—Cu1—Cl1	145.15 (6)
C10—C9—C8	120.5 (3)	O1W—Cu1—Cl2	88.86 (6)
C9—C10—C11	120.3 (3)	N2—Cu1—Cl2	94.66 (6)
C10—C11—C12	120.0 (3)	N1—Cu1—Cl2	106.80 (6)
C7—C12—C11	119.6 (3)	Cl1—Cu1—Cl2	108.04 (3)

### Hydrogen-bond geometry (Å, º)

**Table:**

*D*—H···*A*	*D*—H	H···*A*	*D*···*A*	*D*—H···*A*
O1*W*—H1*A*···O2*W*^i^	0.85 (1)	1.80 (1)	2.640 (3)	171 (3)
O1*W*—H1*B*···Cl2^ii^	0.86 (1)	2.35 (2)	3.1740 (19)	162 (3)
O2*W*—H2*A*···Cl2	0.85 (1)	2.36 (1)	3.199 (2)	171 (3)
O2*W*—H2*B*···Cl1^ii^	0.85 (1)	2.38 (1)	3.215 (2)	167 (3)
C5—H4···O1*W*	0.95 (2)	2.47 (2)	2.999 (3)	115.3 (18)
C6—H5···Cl1^iii^	1.00 (3)	2.56 (3)	3.540 (3)	169 (2)
C8—H6···Cl2	0.87 (3)	2.95 (3)	3.707 (3)	147 (2)
C12—H10···Cl2^iv^	0.95 (3)	2.85 (3)	3.667 (3)	145 (2)

Symmetry codes: (i) *x*, *y*+1, *z*; (ii) −*x*+2, −*y*+1, −*z*+1; (iii) −*x*+3/2, *y*−1/2, −*z*+1/2; (iv) −*x*+3/2, *y*+1/2, −*z*+1/2.
